# Treatment of Insomnia in Forensic Psychiatric Patients: A Randomized Controlled Trial

**DOI:** 10.3390/brainsci15030302

**Published:** 2025-03-12

**Authors:** Maaike Marina Van Veen, Gretha Johanna Boersma, Julie Karsten, Marike Lancel

**Affiliations:** 1 Centre of Expertise on Sleep and Psychiatry, GGZ Drenthe Mental Health Institute, 9404 LA Assen, The Netherlands; 2 Forensic Psychiatric Hospital, GGZ Drenthe Mental Health Institute, 9404 LA Assen, The Netherlands; gretha.boersma@ggzdrenthe.nl (G.J.B.);; 3 Department of Clinical Psychology & Experimental Psychopathology, University of Groningen, 9712 TS Groningen, The Netherlands; j.karsten@rug.nl (J.K.); m.lancel@rug.nl (M.L.)

**Keywords:** insomnia, forensic psychiatry, cognitive behavioral therapy, aggression

## Abstract

**Background:** Insomnia is common in forensic psychiatric patients. Not only does insomnia severely impair general mental health, but it has specifically been associated with poor emotion regulation and self-control, potentially leading to problems in impulsivity, hostility, and even aggression. Cognitive behavioral therapy for insomnia (CBT-I) could therefore be beneficial in this patient group. **Methods:** We conducted a 14-week randomized controlled trial of the effects of cognitive behavioral therapy for insomnia (CBT-I) on sleep, general psychopathology, hostility, impulsivity, and aggression in 31 male forensic psychiatric patients. **Results:** The CBT-I group (n = 11) showed a stronger reduction in self-reported insomnia symptoms and hostility than the waitlist group (n = 11). No differences were found in post-treatment self-reported general psychopathology, impulsivity, or aggression, nor on actigraphy-measured sleep efficiency. **Conclusions:** This study demonstrates the effectiveness of CBT-I in forensic psychiatric patients and indicates the importance of insomnia treatment in this population, especially considering the effect on hostility.

## 1. Introduction

Insomnia disorder, characterized by frequent and persistent difficulties initiating and/or maintaining sleep, affects around 20 to 30% of forensic psychiatric patients [1,2,3]. It is known to have a major impact on general mental health, influencing both the onset and severity of mental health disorders as well as relapses [4,5]. Moreover, disturbed sleep has specifically been associated with poor emotion regulation and self-control [6,7], potentially leading to problems in impulsivity, hostility, and even aggression [8,9]. The association of poor sleep with aggression is confirmed by large meta-analyses and seems to be even more pronounced in those with existing mental health problems [10,11]. Further understanding of this association could be beneficial to forensic psychiatric patients, considering the fact that treatment is often focused on reducing the risk of (repeated) impulsive, hostile, and aggressive behavior.

The treatment of choice for insomnia is cognitive behavioral therapy for insomnia (CBT-I) [12], a multicomponent therapy that has been found feasible and effective in patients with comorbid mental health disorders [13]. Though widely researched in mental health populations, including patients in secure clinical settings [14,15], studies on the secondary impact of CBT-I and related insomnia treatments on irritability, impulsivity, or aggression are scarce. Some small-scale experimental studies show that improving sleep reduces behavioral problems and aggression [16,17]. These results suggest that insomnia treatment might be a promising method to alleviate insomnia and also indirectly prevent aggressive behavior in populations at high risk for such behavior, such as forensic psychiatric patients.

Despite the high prevalence of insomnia, sleep-improving interventions have hardly been studied in forensic patients [8]. In the multifactorial and complex treatments that are often required in forensic psychiatry, insomnia may have been regarded as merely a symptom of psychopathology that would subside upon treatment of the mental health disorder, thus neglecting the necessity of directly targeting sleep [2,5]. In addition, this complex patient group is particularly difficult to motivate for and adhere to specific interventions, partly because of the often compulsory nature of their treatment [18]. Though poor sleep at the start of forensic treatment was shown to be associated with increased aggression over time [19], no studies to date have directly addressed whether CBT-I is effective for improving sleep, overall mental health, and/or reducing aggression and related symptoms in the forensic psychiatric population.

The current study therefore aims to investigate the effects of the first-choice insomnia treatment, CBT-I, on sleep, general level of psychopathology, hostility, impulsivity, and aggression in forensic psychiatric patients.

## 2. Materials and Methods

### 2.1. Participants and Design

Recruited patients were males between 18 and 65 years of age who either had committed or were at risk for committing a crime and were undergoing treatment in forensic psychiatry for their psychiatric disorder. Treatment took place in a secure hospital or outpatient care setting and was often court-ordered and involuntary. The treating physician or psychologist first identified eligible patients with insomnia complaints who were open to being approached for study participation. These patients were subsequently screened for inclusion by the research team. All patients were suffering from insomnia disorder according to the 3rd edition of the International Classification of Sleep Disorders (ICSD-3) criteria [20], as assessed by a clinical interview and Sleep Diagnosis Questionnaire (SDQ) [21]. Insomnia disorder is defined in the ICSD-3 as frequent (≥3 nights per week) and long-lasting (≥3 months) difficulties initiating and/or maintaining sleep causing significant daytime impairment, that are not better explained by other sleep disorders. Those with a high likelihood of other sleep disorders, such as obstructive sleep apnea or restless legs syndrome, were excluded from this study and appropriately referred for further medical examination. Two items of a risk assessment instrument, the Historical Clinical Future-30 (Dutch abbreviation: HKT-30) [22], were used to identify clinician-rated impulsivity and hostility. Patients scoring at least one on either of these items were included, with about 4% of eligible patients not meeting this criterion. All participants enrolled in this study provided written informed consent.

A randomized controlled intervention study was performed, comparing CBT-I to a waitlist condition in addition to ongoing treatment as usual. Participants were allocated using an online block randomization application (www.sealedenvelope.com, accessed on 1 June 2017). The CBT-I protocol consisted of 7 weekly 1 h sessions encompassing treatment components such as sleep hygiene, stimulus control, sleep restriction, relaxation techniques, and cognitive therapy [23]. The interventions were equally divided between two psychologists with previous experience in practicing CBT-I, who were instructed to follow the original protocol [23] as strictly as possible. Those initially assigned to the waitlist condition were offered CBT-I treatment upon study completion after 14 weeks. Measurements were conducted at baseline, directly after treatment (7 weeks), and at follow-up (14 weeks). The employee assessing all outcome measurements was blind to participants’ allocation. This study was approved by the Medical Ethical Committee of Isala Clinics (Zwolle, The Netherlands) and registered in the International Clinical Trials Registry Platform (NL7943).

### 2.2. Data Collection

Information on psychiatric diagnoses and current use of (hypnotic) medication was extracted from the medical files. Insomnia complaints were assessed by the Insomnia Severity Index (ISI) [24], of which the total score is indicative of mild (≥8) or severe (≥15) insomnia. A decrease of minimally 8 points is considered to be a clinically relevant treatment response [25]. General level of psychopathology was measured by the Symptom Checklist 90 (SCL-90) [26], assessing a wide variety of symptoms including affect, somatic symptoms, interpersonal sensitivity, and sleep complaints. Several subscales can be computed, of which the hostility subscale, measuring irritability, anger, and aggression, was of most interest to our research question. We used a corrected total SCL-90 score by leaving out the 3 sleep items, aiming to discern the sleep intervention effect on general psychopathology apart from sleep. Self-reported impulsivity and aggression were measured by the Barratt Impulsiveness Scale (BIS) [27,28] and Aggression Questionnaire (AQ) [29,30], respectively. Wrist actigraphy (Actiwatch Spectrum Plus, Phillips Respironics 2014) was performed to obtain an objective measure of sleep, using Actiware version 5.70 software (Royal Phillips Electronics N.V. 2012). We used sleep efficiency, i.e., the ratio between the time spent asleep and the total time in bed dedicated to sleep, which is a widely used general measure of sleep quality [31]. Additionally, we aimed to perform two neuropsychological tests to objectively assess the level of impulsivity (Stop Signal Task) [32] and risk taking (Iowa Gambling Task) [33]. Unfortunately, we eventually decided to not further analyze the data obtained by these tests because of high numbers of missing values.

### 2.3. Statistical Analysis

Data were reported as mean and standard deviation for all continuous variables, whereas categorical variables were reported as percentages. The effects of treatment on outcome measures were investigated with repeated measures ANOVA (rmANOVA) with time as a within-subject factor and group as a between-subject factor, adjusting for age and for baseline values to correct for possible group differences despite randomization. A *p* value < 0.05 was considered statistically significant. All analyses were carried out using SPSS statistical software, version 26 (IBM SPSS Statistics for Windows, Version 26.0. IBM Corp., Armonk, NY, USA).

## 3. Results

**Figure 1:**
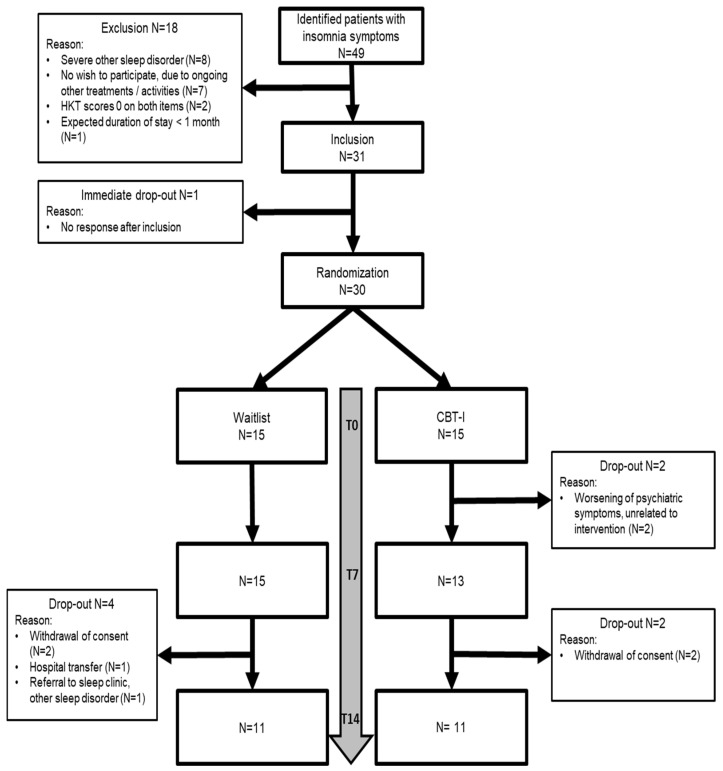
Flow of participants through this study.

A total of 31 patients were included in this study, with 11 participants completing the follow-up measurements in each arm. The flow of participants through this study is shown in Figure 1.

The mean age was 38.5 ± 12.3 years old (range 19–63). Common psychiatric diagnoses varied from personality disorders (predominantly antisocial and/or borderline, or traits thereof (71.0%)) and any kind of substance abuse disorder (38.7%) to attention deficit hyperactivity disorder (ADHD) (22.6%), paraphilic disorder (22.6%), and periodic explosive disorder (22.6%). Most participants had two or more psychiatric diagnoses. About half of the participants (51.6%) used sleep medication at the time of enrollment in this study. The baseline characteristics of enrolled participants from both groups are shown in Table 1.

**Figure 2:**
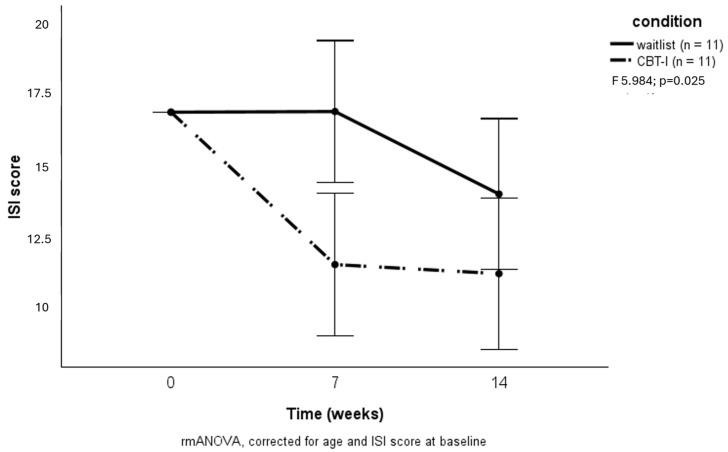
Effects of CBT-I on insomnia severity.

Participants receiving CBT-I treatment showed a significant decrease in insomnia symptoms compared to those in the waitlist condition (F = 5.984; *p* = 0.025) (Figure 2).

**Figure 3:**
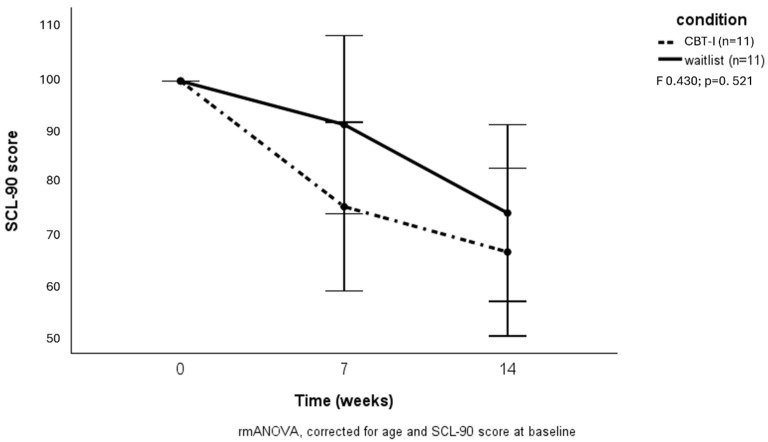
Effects of CBT-I on general psychopathology.

**Figure 4:**
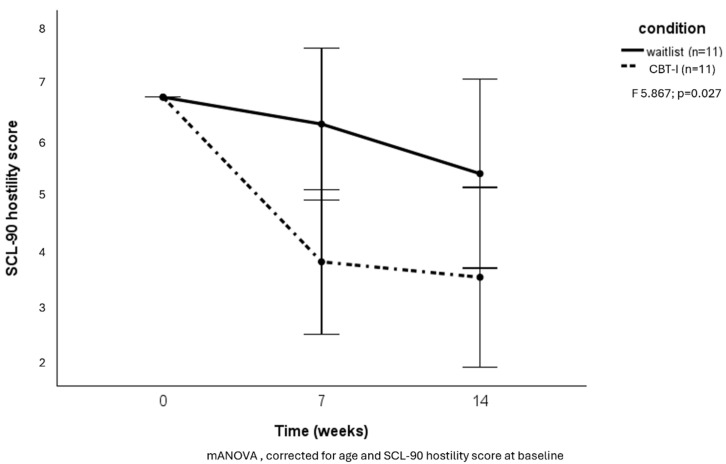
Effects of CBT-I on hostility.

A clinically relevant response (minimally eight points decrease in ISI score) after 14 weeks was reached in five of those receiving CBT-I (33%) versus one participant in the waitlist condition (6.7%) (Χ^2^ = 3.33; *p* = 0.07). No difference was found between groups regarding a decrease in general psychopathology (Figure 3). However, the hostility subscale of the SCL-90 showed a significant decrease in the CBT-I group (F = 5.867; *p* = 0.03) (Figure 4).

We found no differences between groups regarding changes in actigraphy-measured sleep efficiency (F = 1.523; *p* = 0.236), self-reported impulsivity (F = 0.424; *p* = 0.658), and self-reported aggression (F = 0.477; *p* = 0.624).

Post hoc analyses revealed a significant difference between groups from baseline to week 7 in insomnia symptoms (F = 5.952; *p* = 0.023) and hostility (F = 7.518; *p* = 0.012), but not from week 7 to week 14 (F = 2.243; *p* = 0.152 for insomnia and F = 0.371; *p* = 0.551 for hostility).

## 4. Discussion

Insomnia is associated with poor emotion regulation and self-control, posing sleep interventions as an interesting opportunity to reduce hostility, impulsivity, and aggression in high-risk populations, such as forensic psychiatric patients. Our study aimed to evaluate the effects of targeted insomnia treatment on sleep, general psychopathology, hostility, impulsivity, and aggression in forensic psychiatric patients.

CBT-I proved to be effective in reducing insomnia symptoms when compared to the waitlist group. Dropout rates were similar in both study arms, demonstrating the feasibility of CBT-I in forensic patients. Participants receiving the intervention showed an immediate decrease in insomnia symptoms, which was sustained during follow-up. Those in the waitlist condition also showed some improvement during follow-up, in line with both previous evidence and our clinical experience that insomnia symptoms fluctuate [34]. Notably, one-third of CBT-I participants reached a clinically relevant reduction in insomnia symptoms versus just one of the participants in the wait-list condition, a rather large difference that was not statistically significant, likely due to our small sample size. The improvement in sleep quality in the CBT-I group was not observed in actigraphy-measured sleep efficiency. These findings are comparable to Dewa et al., who also found positive effects on self-reported insomnia in a study among prisoners, but not in actigraphy-measured sleep [35]. Differences in the subjective experience of sleep and what can be objectively measured are well known in sleep research in general, posing a challenge to both choice of measurements and interpretation of results [36]. As for insomnia, the patients’ perception of sleep quality is mandatory to both diagnosis and treatment; we believe subjective evaluation should be the most telling outcome.

Though no difference in reduction in general psychopathology was found, we found a striking reduction in hostility in the CBT-I group. This finding is of great clinical importance because hostility is a known risk factor for (repeated) offenses in forensic psychiatric patients, and irritability and anger, features of the hostility subscale, are key symptoms on which treatment is focused [22]. As all participants, both in the intervention and waitlist groups, were receiving treatment as usual parallel to this study, the reduction in hostility in the CBT-I group can be assumed to be a direct effect of the alleviation of insomnia symptoms. Hypothetically, this could be due to enhanced emotion regulation and cognitive reappraisal of negative perceptions [9]. Another explanation may be that the increase in sleep quality improves both adherence to and effectiveness of the ongoing forensic treatment in general [37]. We could not confirm a reduction in self-reported impulsivity or aggression, possibly because the questionnaires we used measure more static and less dynamic constructs.

The recruitment of participants proved to be a challenging process as sleep disorders are not systematically assessed in forensic psychiatric patients, despite the previously found high prevalence of insomnia of 20–30% in this population [1,2,3]. As forensic treatment focuses on reducing aggression by identifying possible risk factors, the incorporation of sleep in standard forensic diagnostic and risk assessment instruments could be of great value. This has already been explored in general psychiatric intensive care, where the addition of a sleep variable to daily risk assessments improved the prediction of aggressive incidents [38]. The patients who were eventually identified and approached for this intervention study showed high willingness to enroll, demonstrated by only 14.3% declining consent after being further informed about participation. To our clinical experience, this is indicative of the burden of insomnia: patients are often very motivated to alleviate their insomnia symptoms. Still, the small number of participants is an important limitation of our study, limiting our ability to correct for possible confounders and warranting modesty in the interpretation of results. Another limitation is the naturalistic approach, focusing on the additional effects of CBT-I and thus accepting the fact that TAU comprised interventions and personal circumstances we could not account for. Inherent to such a design is a reduction in certainty that the group difference found is solely caused by the intervention. For example, we have information on the use of sleep medication (yes/no) upon inclusion but not at follow-up. However, at baseline we did not find any difference in sleep medication use (Table 1). Though randomization of participants theoretically reduces the risk that known and unknown factors are different between groups, accepting ongoing care as usual may be regarded as a methodological disadvantage. On the other hand, it may provide a more realistic and generalizable view of the effects of the intervention.

Though this study is small in size, it is unique because the worldwide first-choice protocolized insomnia treatment, CBT-I, had not been investigated in a forensic psychiatric population before. Our results underline the importance of integrating diagnostic assessment and treatment of sleep disorders in forensic psychiatry, possibly with population-specific adjustments [2,35]. In our view, it would be interesting for future researchers to investigate how the effects of sleep interventions and their influence on hostility translate to the actual occurrence of aggressive behavior and whether such effects are a direct result of changes in sleep quality or rather are due to enhanced effectiveness of general forensic treatment. Either way, sleep interventions are likely to be important additions to forensic psychiatric care.

## 5. Conclusions

CBT-I treatment is feasible as well as effective in this forensic psychiatric population. Both self-reported insomnia symptoms and hostility persistently improved following treatment. Although small, this study supports existing evidence that CBT-I can and should be applied in complex patient populations. Especially in forensic psychiatry, targeted treatment of insomnia may constitute a viable option to indirectly reduce the risk of aggressive behavior. This calls for solid and broad implementation of the assessment and management of sleep problems in forensic treatment settings.

## Figures and Tables

**Table 1:** Baseline characteristics (mean ± SD or n (%)) of enrolled participants (waitlist group and CBT-I group).

	Waitlist	CBT-I
Age	38.1 ± 12.0	38.9 ± 13.0
Use of sleep medication	7 (46.7%)	9 (56.3%)
Psychiatric diagnoses		
Attention deficit hyperactivity disorder	4 (25.0%)	3 (20.0%)
Periodic explosive disorder	5 (31.3%)	2 (13.3%)
Substance use disorder	6 (37.5%)	6 (40%)
Borderline personality disorder	3 (18.8%)	2 (13.3%)
Antisocial personality disorder	3 (18.8%)	3 (20.0%)
Personality disorder NOS	3 (18.8%)	8 (53.3%)
Post-traumatic stress disorder	0 (0.0%)	5 (33.3%)
Paraphilia/pedophilia	3 (18.8%)	4 (26.7%)
Outcome measures		
Insomnia severity (ISI)	15.8 ± 4.3	18.0 ± 3.6
General psychopathology (SCL-90)	102.4 ± 55.9	108.7 ± 48.4
Hostility (SCL-90 subscale)	7.6 ± 5.9	7.3 ± 4.4
Impulsivity (BIS)	73.1 ± 12.7	70.2 ± 10.1
Aggression (AQ)	85.0 ± 23.1	95.9 ± 14.5
Sleep efficiency (actigraphy)	75.6 ± 16.3	79.0 ± 5.0

ISI = Insomnia Severity Index; SCL-90 = Symptom Checklist-90; BIS = Barratt Impulsiveness Scale; AQ = Aggression Questionnaire.

## Data Availability

The datasets presented in this article are not readily available because of privacy reasons. Requests to access the datasets should be directed to gretha.boersma@ggzdrenthe.nl.

## References

[1] Kamphuis J., Karsten J., de Weerd A., Lancel M. (2013). Sleep disturbances in a clinical forensic psychiatric population. Sleep Med..

[2] Mijnster T., Boersma G., Engberts J., Vreugdenhil-Becherer L., Keulen-de Vos M., de Vogel V., Bulten E., Lancel M. (2022). Lying awake in forensic hospitals: A multicenter, cross-sectional study on the prevalence of insomnia and contributing factors in forensic psychiatric patients. J. Forensic Psychiatry Psychol..

[3] Van Veen M.M., Karsten J., Lancel M. (2017). Poor sleep and its relation to impulsivity in patients with antisocial or borderline personality disorders. Behav. Med..

[4] Baglioni C., Nanovska S., Regen W., Spiegelhalder K., Feige B., Nissen C., Reynolds C.F., Riemann D. (2016). Sleep and mental disorders: A meta-analysis of polysomnographic research. Psychol. Bull..

[5] Freeman D., Sheaves B., Waite F., Harvey A.G., Harrison P.J. (2020). Sleep disturbance and psychiatric disorders. Lancet Psychiatry.

[6] Walker M.P. (2009). The role of sleep in cognition and emotion. Ann. N. Y. Acad. Sci..

[7] Palmer C.A., Alfano C.A. (2017). Sleep and emotion regulation: An organizing, integrative review. Sleep Med. Rev..

[8] Kamphuis J., Meerlo P., Koolhaas J.M., Lancel M. (2012). Poor sleep as a potential causal factor in aggression and violence. Sleep Med..

[9] Krizan Z., Herlache A.D. (2016). Sleep disruption and aggression: Implications for violence and its prevention. Psychol. Violence.

[10] Van Veen M.M., Lancel M., Beijer E., Remmelzwaal S., Rutters F. (2021). The association of sleep quality and aggression: A systematic review and meta-analysis of observational studies. Sleep Med. Rev..

[11] Van Veen M.M., Lancel M., Şener O., Verkes R.J., Bouman E.J., Rutters F. (2022). Observational and experimental studies on sleep duration and aggression: A systematic review and meta-analysis. Sleep Med. Rev..

[12] Riemann D., Baglioni C., Bassetti C., Bjorvatn B., Dolenc Groselj L., Ellis J.G., Espie C.A., Garcia-Borreguero D., Gjerstad M., Gonçalves M. (2017). European guideline for the diagnosis and treatment of insomnia. J. Sleep Res..

[13] Hertenstein E., Trinca E., Wunderlin M., Schneider C.L., Züst M.A., Fehér K.D., Su T., Straten A.V., Berger T., Baglioni C. (2022). Cognitive behavioral therapy for insomnia in patients with mental disorders and comorbid insomnia: A systematic review and meta-analysis. Sleep Med. Rev..

[14] Gardiner P.M., Kinnafick F., Breen K.C., Girardi A., Hartescu I. (2022). Behavioural, medical & environmental interventions to improve sleep quality for mental health inpatients in secure settings: A systematic review & meta-analysis. J. Forensic Psychiatry Psychol..

[15] Mijnster T., Boersma G.J., Meijer E., Lancel M. (2022). Effectivity of (personalized) cognitive behavioral therapy for insomnia in mental health populations and the elderly: An overview. J. Pers. Med..

[16] Blake M.J., Snoep L., Raniti M., Schwartz O., Waloszek J.M., Simmons J.G., Murray G., Blake L., Landau E.R., Dahl R.E. (2017). A cognitive-behavioral and mindfulness-based group sleep intervention improves behavior problems in at-risk adolescents by improving perceived sleep quality. Behav. Res. Ther..

[17] Haynes P.L., Bootzin R.R., Smith L., Cousins J., Cameron M., Stevens S. (2006). Sleep and aggression in substance-abusing adolescents: Results from an integrative behavioral sleep-treatment pilot program. Sleep.

[18] Brunner F., Neumann I., Yoon D., Rettenberger M., Stück E., Briken P. (2019). Determinants of dropout from correctional offender treatment. Front. Psychiatry.

[19] Van Veen M.M., Rutters F., Spreen M., Lancel M. (2020). Poor sleep quality at baseline is associated with increased aggression over one year in forensic psychiatric patients. Sleep Med..

[20] Sateia M.J. (2014). International classification of sleep disorders-third edition: Highlights and modifications. Chest.

[21] Sweere Y., Kerkhof G.A., De Weerd A.W., Kamphuisen HA C., Kemp B., Schimsheimer R.J. (1998). The validity of the dutch sleep disorders questionnaire (sdq). J. Psychosom. Res..

[22] Projectgroup Risk Assessment in Forensic Psychiatry (2003). Manual HKT-30 Version 2002, Risk Assessment in Forensic Psychiatry.

[23] Perlis M.L., Jungquist C.R., Smith M.T., Posner D.A. (2005). Cognitive Behavioral Treatment of Insomnia—A Session-by-Session Guide.

[24] Bastien C.H., Vallieres A., Morin C.M. (2001). Validation of the insomnia severity index as an outcome measure for insomnia research. Sleep Med..

[25] Morin C.M., Belleville G., Belanger L., Ivers H. (2011). The insomnia severity index: Psychometric indicators to detect insomnia cases and evaluate treatment response. Sleep.

[26] Derogatis L.R., Melisaratos N. (1983). The brief symptom inventory: An introductory report. Psychol. Med..

[27] Patton J.H., Stanford M.S., Barratt E.S. (1995). Factor structure of the barratt impulsiveness scale. J. Clin. Psychol..

[28] Vasconcelos A.G., Malloy-Diniz L., Correa H. (2012). Systematic review of psychometric proprieties of barratt impulsiveness scale version 11 (BIS-11). Clin. Neuropsychiatry J. Treat. Eval..

[29] Buss A.H., Perry M. (1992). The aggression questionnaire. J. Personal. Soc. Psychol..

[30] Morren M., Meesters C. (2002). Validation of the dutch version of the aggression questionnaire in adolescent male offenders. Aggress. Behav..

[31] Littner M., Kushida C.A., Anderson W.M., Bailey D., Berry R.B., Davila D.G., Hirshkowitz M., Kapen S., Kramer M., Loube D. (2003). Practice parameters for the role of actigraphy in the study of sleep and circadian rhythms: An update for 2002. Sleep.

[32] Logan G.D., Cowan W.B., Davis K.A. (1984). On the ability to inhibit simple and choice reaction time responses: A model and a method. Journal of Experimental Psychology. Hum. Percept. Perform..

[33] Toplak M.E., Sorge G.B., Benoit A., West R.F., Stanovich K.E. (2010). Decision-making and cognitive abilities: A review of associations between iowa gambling task performance, executive functions, and intelligence. Clin. Psychol. Rev..

[34] Morin C.M., Bélanger L., LeBlanc M., Ivers H., Savard J., Espie C.A., Mérette C., Baillargeon L., Grégoire J.P. (2009). The natural history of insomnia: A population-based 3-year longitudinal study. Arch. Intern. Med..

[35] Dewa L.H., Thibaut B., Pattison N., Campbell S.J., Woodcock T., Aylin P., Archer S. (2024). Treating insomnia in people who are incarcerated: A feasibility study of a multicomponent treatment pathway. Sleep Adv. J. Sleep Res. Soc..

[36] Kaplan K.A., Hirshman J., Hernandez B., Stefanick M.L., Hoffman A.R., Redline S., Ancoli-Israel S., Stone K., Friedman L., Zeitzer J.M. (2017). When a gold standard isn’t so golden: Lack of prediction of subjective sleep quality from sleep polysomnography. Biol. Psychol..

[37] Dolsen M.R., Soehner A.M., Morin C.M., Bélanger L., Walker M., Harvey A.G. (2017). Sleep the night before and after a treatment session: A critical ingredient for treatment adherence?. J. Consult. Clin. Psychol..

[38] Langsrud K., Vaaler A., Morken G., Kallestad H., Almvik R., Palmstierna T., Güzey I.C. (2019). The predictive properties of violence risk instruments may increase by adding items assessing sleep. Front. Psychiatry.

